# Medical errors in Kenya

**Published:** 2019-09-10

**Authors:** Nyawira Mwangi

**Affiliations:** 1 Principal Lecturer: Ophthalmology Programs, Kenya Medical Training College, Nairobi and Research Fellow: London School of Hygiene and Tropical Medicine, UK.

When there is a medical error, we follow these steps:

1. If the error is noted at the time of surgery or soon after, provide emergency care that might save the vision or eye.
2. Communicate with the patient, explain what has happened, what care will be provided (perhaps waiving costs), and the likely outcomes.
3. Have a meeting with the team to identify why the error occurred and what should be done to prevent it from happening again. The challenge here is that the meeting has to be specific and candid enough to identify the root cause, but at the same time avoid the ‘quick fix’ of simply identifying who is to be blamed.
4. Document the entire process and make a report.
5. If the hospital has a morbidity meeting, encourage staff members to present the case.
6. Implement measures to prevent this in future, such as additional medication checks, training or staffing.
7. Anticipate legal or professional actions from this event.

